# Mitochondrial genomes of two diplectanids (Platyhelminthes: Monogenea) expose paraphyly of the order Dactylogyridea and extensive tRNA gene rearrangements

**DOI:** 10.1186/s13071-018-3144-6

**Published:** 2018-11-20

**Authors:** Dong Zhang, Wen X. Li, Hong Zou, Shan G. Wu, Ming Li, Ivan Jakovlić, Jin Zhang, Rong Chen, Gui T. Wang

**Affiliations:** 1 Key Laboratory of Aquaculture Disease Control, Ministry of Agriculture, and State Key Laboratory of Freshwater Ecology and Biotechnology, Institute of Hydrobiology, Chinese Academy of Sciences, Wuhan, 430072 People’s Republic of China; 2 University of Chinese Academy of Sciences, Beijing, People’s Republic of China; 3 Bio-Transduction Lab, Biolake, Wuhan, 430075 People’s Republic of China

**Keywords:** Phylogenomics, Gene rearrangement, Molecular markers, Gene loss, Pseudo tRNA gene tandem array

## Abstract

**Background:**

Recent mitochondrial phylogenomics studies have reported a sister-group relationship of the orders Capsalidea and Dactylogyridea, which is inconsistent with previous morphology- and molecular-based phylogenies. As Dactylogyridea mitochondrial genomes (mitogenomes) are currently represented by only one family, to improve the phylogenetic resolution, we sequenced and characterized two dactylogyridean parasites, *Lamellodiscus spari* and *Lepidotrema longipenis*, belonging to a non-represented family Diplectanidae.

**Results:**

The *L. longipenis* mitogenome (15,433 bp) contains the standard 36 flatworm mitochondrial genes (*atp*8 is absent), whereas we failed to detect *trnS*1, *trnC* and *trnG* in *L. spari* (14,614 bp). Both mitogenomes exhibit unique gene orders (among the Monogenea), with a number of tRNA rearrangements. Both long non-coding regions contain a number of different (partially overlapping) repeat sequences. Intriguingly, these include putative tRNA pseudogenes in a tandem array (17 *trnV* pseudogenes in *L. longipenis*, 13 *trnY* pseudogenes in *L. spari*). Combined nucleotide diversity, non-synonymous/synonymous substitutions ratio and average sequence identity analyses consistently showed that *nad*2, *nad*5 and *nad*4 were the most variable PCGs, whereas *cox*1, *cox*2 and *cytb* were the most conserved. Phylogenomic analysis showed that the newly sequenced species of the family Diplectanidae formed a sister-group with the Dactylogyridae + Capsalidae clade. Thus Dactylogyridea (represented by the Diplectanidae and Dactylogyridae) was rendered paraphyletic (with high statistical support) by the nested Capsalidea (represented by the Capsalidae) clade.

**Conclusions:**

Our results show that *nad*2, *nad*5 and *nad*4 (fast-evolving) would be better candidates than *cox*1 (slow-evolving) for species identification and population genetics studies in the Diplectanidae. The unique gene order pattern further suggests discontinuous evolution of mitogenomic gene order arrangement in the Class Monogenea. This first report of paraphyly of the Dactylogyridea highlights the need to generate more molecular data for monogenean parasites, in order to be able to clarify their relationships using large datasets, as single-gene markers appear to provide a phylogenetic resolution which is too low for the task.

**Electronic supplementary material:**

The online version of this article (10.1186/s13071-018-3144-6) contains supplementary material, which is available to authorized users.

## Background

Monogeneans of the family Diplectanidae (Dactylogyridea: Dactylogyrinea) are parasites found on the gills of (mostly) marine perciform fishes [1]. The family comprises approximately 250 species and is mainly studied for the adverse health effects these parasites cause to the hosts; the fixation of their opisthaptors on the gills causes haemorrhages and a white mucoid exudate, which often leads to secondary fungal, bacterial and/or viral infections [2, 3]. An example is *Diplectanum aequans* (Wagener, 1857), which can cause high mortality of juvenile European sea bass in the Mediterranean aquaculture [2].

Traditionally used phylogenetic markers, morphology and single-genes, are often not suitable for resolving evolutionary history with high confidence; morphological traits can be homoplastic, which often causes taxonomic and phylogenetic artifacts [4–6] and, due to the small amount of information (phylogenetic signal) they carry, single-gene molecular markers may have limited resolving power [7]. This is reflected in the unresolved phylogeny of Monogenean parasites [8–11]. Specifically, studies based on spermatozoal ultrastructural characters [12, 13], the *18S rRNA* gene [9–11], and a combination of three unlinked nuclear genes [5], supported a phylogenetically closer relationship between monogenean orders Capsalidea and Gyrodactylidea than (either of the two) to the Dactylogyridea. However, 66 homologous series of morphological characters resolved the Gyrodactylidea and Dactylogyridea as sister groups [14]. Therefore, molecular markers carrying more powerful phylogenetic signals are needed to resolve their phylogenetic relationships with high resolution. Mitogenome is a good candidate marker, with an approximately ten times larger nucleotide alignment length than commonly used single-gene molecular markers (ITS, *18S* and *28S rRNA*). Although their applicability for studies of the Neodermata is still hampered by their relative scarcity, they are increasingly used in population genetics [15], phylogenetics [16, 17] and diagnostics [7, 18] of parasitic flatworms, despite this limitation. Intriguingly, recent researches [4, 19, 20] relying on the mitochondrial (mt) phylogenomics approach consistently resolved the Dactylogyridea and Capsalidea as sister-groups, thereby further complicating phylogenetic hypotheses for the three aforementioned orders.

As the resolution power of mitochondrial genomics is still limited by the very low number of sequenced monogenean mitogenomes available, where many taxonomic categories remain poorly represented or unrepresented (only one dactylogyridean family represented), we sequenced and characterized two complete mitochondrial genomes belonging to a non-represented dactylogyridean family, the Diplectanidae: *Lamellodiscus spari* (Zhukov, 1970) and *Lepidotrema longipenis* (Yamaguti, 1934), collected from the gills of two marine fish species*.* Their availability shall enable us to employ mitochondrial phylogenomics to investigate relationships of these three orders with improved resolution.

## Methods

### Specimen collection and identification

According to the records in Zhang et al. [21], we searched for diplectanid parasites by exploring fish markets in several coastal cities in the southern China. *Lepidotrema longipenis* was obtained from *Terapon jarbua* (Forsskål, 1775) (Perciformes: Terapontidae) bought at a local market in Zhanjiang city, Guangdong Province (21°15'5"-21°15'16"N, 110°23'46"-110°24'12"E), on the 18th June 2016. *Lamellodiscus spari* was obtained from the black sea bream *Acanthopagrus schlegelii* (Bleeker, 1854) (Perciformes: Sparidae) caught by fishermen in Daya Bay, Guangdong Province (22°42'58"-22°42'56"N, 114°32'16"-114°32'25"E) on the 10th July 2017. Discriminative morphological characteristics for the Diplectanidae are their opisthaptor equipped by three transversal bars connected to two pairs of central hooks, 14 marginal hooks and accessory adhesive organ (lamellodisc or squamodisc) that can be present or absent [22, 23]. Parasites were further morphologically identified to the genus level as described in Domingues & Boeger [23], and to the species level under a light microscope according to the traits described in Ogawa & Egusa [24] for *L. spari*, and the traits described in Zhang et al. [21] for *L. longipenis*. Additionally, to confirm the taxonomic identity, the *28S rRNA* gene was amplified using universal primers [25] (Additional file 1: Dataset S1); both species share a very high identity of 99.6% with corresponding conspecific homologs available in GenBank: 744/747 identical bp for *L. longipenis* (EF100563), and (837/840) for *L. spari* (DQ054823). All sampled and identified parasites were first washed in 0.6% saline and then stored in 100% ethanol at 4 °C.

### DNA extraction, amplification and sequencing

Due to the small size of these parasites, we used two kinds of genomic DNA to ensure a sufficient amount for amplification and sequencing, both extracted using a TIANamp MicroDNA Kit (Tiangen Biotech, Beijing, China): mixture DNA (20 parasite specimens) and individual DNA (a single parasite specimen). Mixture DNA was first used to amplify the whole mitogenome. First, we selected 14 monogenean mitogenomes from GenBank, aligned them using ClustalX [26], and designed degenerate primer pairs (Additional file 1: Dataset S1) matching the generally conserved regions of mitochondrial genes (*16S*, *12S*, *cox*1, *cox*2, *nad*1, *nad*4 and *cytb*). On the basis of these obtained fragments, specific primers were then designed using Primer Premier 5 [27], and the remaining mitogenome was amplified and sequenced in several PCR steps (Additional file 1: Dataset S1). Both mitogenomes were amplified exactly following the procedures previously described [4, 17, 19, 28]; detailed PCR conditions are provided in Additional file 1: Dataset S1. PCR products were sequenced bi-directionally using both degenerate and specific primers mentioned above on an ABI 3730 automatic sequencer (Sangon, Shanghai, China) using the Sanger method. During the sequencing we paid close attention to chromatograms, carefully examining them for double peaks, or any other sign of the existence of two different sequences. A BLAST [29] check was used to confirm that all amplicons are the actual target sequences. To address the possibility of intraspecific sequence variation present in the mixture DNA, we then used individual DNA and long-range PCR to verify the obtained sequences (primers used are listed in Additional file 1: Dataset S1). If we found two different sequences, we used the DNA extracted from a single individual to infer the mitogenomic sequence, thereby ensuring that each sequence belongs to a single specimen.

### Sequence annotation and analyses

Both mitogenomes were assembled and annotated following a previously described procedure [4, 17, 19, 28] using DNAstar v.7.1 software [30], MITOS [31], ARWEN [32] and DOGMA [33] web tools, so detailed methodology is provided in Additional file 1: Dataset S1. Codon usage and relative synonymous codon usage (RSCU) for 12 protein-encoding genes (PCGs) of the two studied diplectanids, two dactylogyrids (*Dactylogyrus lamellatus* Achmerov, 1952 and *Tetrancistrum nebulosi* Young, 1967) and three capsalids (*Neobenedenia melleni* MacCallum, 1927, *Benedenia seriolae* Yamaguti, 1934 and *B. hoshinai* Ogawa, 1984) were computed and sorted using MitoTool [34] (an in-house GUI-based software), and finally the RSCU figure drawn using the ggplot2 [35] plugin. Non-synonymous (dN) / synonymous (dS) mutation rate ratios among the 12 PCGs of the two studied diplectanid mitogenomes were calculated with DnaSP v.5 [36]. The same software was also employed to conduct the sliding window analysis: a sliding window of 200 bp and a step size of 20 bp were implemented to estimate the nucleotide divergence Pi between the mitogenomes of *L. longipenis* and *L. spari*. Tandem Repeats Finder [37] was employed to find tandem repeats in the long non-coding regions (LNCR), and their secondary structures were predicted by Mfold software [38]. Rearrangement events in the mitogenomes and pairwise comparisons of gene orders of all 20 available monogeneans were calculated with the CREx program [39] using the common intervals measurement. Due to limitations of the CREx algorithm, and to facilitate comparative analyses of gene orders, we provisionally added the gene block *trnS1-trnC-trnG* to the gene order sequence of *L. spari*, corresponding to the position where these genes were found in *L. longipenis* (between *nad*5 and *cox*3). Genetic distances (identity) among mitogenomic sequences were calculated with the “DistanceCalculator” function in Biopython using the “identity” model.

### Phylogenetic analyses

Phylogenetic analyses were conducted using the two newly sequenced diplectanid mitogenomes and all 18 monogenean mitogenomes available in GenBank (5th May 2018). Two species of the order Tricladida, *Crenobia alpina* (Dana, 1766) (KP208776) and *Obama* sp. (NC_026978), were used as outgroups, thus making a total of 22 mitogenomes (Additional file 2: Table S1). Two datasets were used for phylogenetic analysis: amino acid alignment of 12 protein-coding genes (PCGAA) and codon-based alignment of nucleotide sequences of 12 protein-coding genes + secondary structure alignment of 22 tRNAs and 2 rRNAs (PCGRT). As data processing was conducted as previously described [4, 17, 19, 28, 40], using MitoTool, MAFFT [41] and Gblocks [42]; details are given in Additional file 1: Dataset S1. The heterogeneity of sequence divergence within data sets was analyzed using AliGROOVE [43], wherein indels in nucleotide dataset were treated as ambiguity, and a BLOSUM62 matrix was used for amino acids. Best partitioning scheme and evolutionary models were selected using PartitionFinder2 [44], with greedy algorithm and AICc criterion. Phylogenetic analyses were conducted using two different algorithms: maximum likelihood (ML) and Bayesian inference (BI). Based on the Akaike information criterion implemented in ProtTest [45], MTART+I+G+F was chosen as the optimal evolutionary model for the downstream phylogenetic analyses. Under this optimal model and partition model, ML analysis was conducted in RAxML [46] using a ML+rapid bootstrap (BS) algorithm with 1000 replicates. Bayesian inference analyses with the empirical MTART model were conducted using PhyloBayes (PB) MPI 1.5a [47]. For each analysis, two MCMC chains were run after the removal of invariable sites from the alignment, and the analysis was stopped when the conditions considered to indicate a good run (according to the PhyloBayes manual) were reached: maxdiff < 0.1 and minimum effective size > 300. Phylograms and gene orders were visualized and annotated by iTOL [48] with the help of several dataset files generated by MitoTool, as described in our recent papers [4, 17].

## Results and discussion

### Genome organization and base composition

**Fig. 1:**
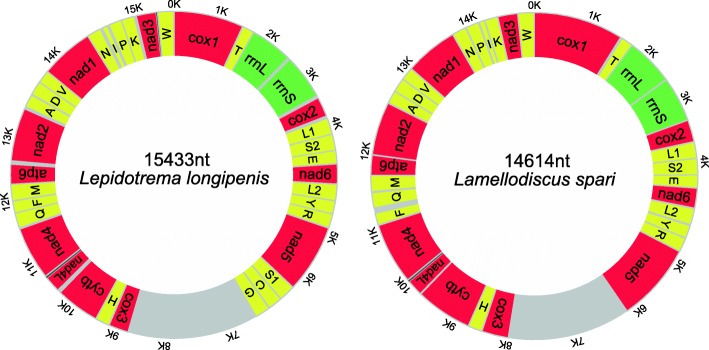
Visual representation of the circular mitochondrial genomes of *Lepidotrema longipenis* and *Lamellodiscus spari*. *Key*: Red, protein-coding genes; yellow, tRNAs; green, rRNAs; grey, non-coding regions

The full circular mitochondrial genome of *L. longipenis* (GenBank: MH328203), at 15,433 bp, is the longest among the monopisthocotylids characterized so far (Additional file 2: Table S1). The mitogenome of *L. spari* is 14,614 bp in size (GenBank: MH328204). The *L. longipenis* mitogenome contains the standard [49] 36 flatworm mitochondrial genes, including 12 protein-encoding genes (PCGs; *atp*8 is absent), 22 tRNA genes, and two rRNA genes, whereas *trnS*1, *trnC* and *trnG* genes are missing in *L. spari* (Table 1 and Fig. 1). The architecture, gene contents and similarity of orthologous sequences for the two studied mitogenomes are summarized in Table 1. Average sequence similarity of PCGs between the two studied mitogenomes ranged from 55.58 (*nad*5) to 79.45% (*cox*1) (Table 1). In comparison to PCGs, average sequence similarity values between the rRNAs of the two species were higher: 75.43% for *rrnL* and 70.87% for *rrnS*.

**Table 1:** Comparison of the annotated mitochondrial genomes of *Lamellodiscus spari* and *Lepidotrema longipenis*

Gene	Position		Size	Intergenic nucleotides	Codon		Strand	Identity
	From	To			Start	Stop		
*Lepidotrema longipenis*/*Lamellodiscus spari*								
*cox*1	1/1	1548/1557	1548/1557		GTG/GTG	TAA/TAG	H/H	79.45
*trnT*	1553/1581	1609/1641	57/61	4/23			H/H	61.9
*rrnL*	1610/1642	2582/2593	973/952				H/H	73.19
*rrnS*	2609/2599	3328/3335	720/737	26/5			H/H	69.43
*cox*2	3361/3336	3936/3917	576/582	32/-	ATG/ATG	TAG/TAA	H/H	70.79
*trnL*1	3970/3920	4039/3985	70/66	33/2			H/H	56.58
*trnS*2	4040/3985	4104/4050	65/66	-/-1			H/H	70.15
*trnE*	4105/4051	4171/4118	67/68				H/H	77.14
*nad*6	4175/4119	4624/4577	450/459	3/-	ATG/GTG	TAA/TAA	H/H	62.75
*trnL*2	4628/4584	4697/4648	70/65	3/6			H/H	64.29
*trnY*	4700/4662	4767/4727	68/66	2/13			H/H	72.86
*trnR*	4769/4725	4836/4789	68/65	1/-3			H/H	72.06
*nad*5	4840/4790	6366/6193	1527/1404	3/-	ATG/GTG	TAA/TAA	H/H	55.58
*trnS*1	6368/-	6427/-	60/-	1/-			H/-	–
*trnC*	6431/-	6496/-	66/-	3/-			H/-	–
*trnG*	6503/-	6567/-	65/-	6/-			H/-	–
LNCR	6568/6194	8560/7966	1993/1773				H/H	60.71
*cox*3	8561/7967	9214/8617	654/651		ATG/ATG	TAA/TAA	H/H	71.41
*trnH*	9218/8619	9285/8682	68/64	3/1			H/H	76.47
*cytb*	9286/8683	10,374/9771	1089/1089		ATG/ATG	TAG/TAG	H/H	74.93
*nad*4L	10,422/9764	10,670/10,018	249/255	47/-8	ATG/ATG	TAG/TAG	H/H	65.89
*nad*4	10,640/9991	11,857/11,166	1218/1176	-31/-28	ATG/ATG	TAA/TAG	H/H	59.5
*trnQ*	11,874/11,313	11,935/11,375	62/63	16/75			H/H	85.71
*trnF*	11,936/11,173	12,003/11,237	68/65	-/6			H/H	77.94
*trnM*	12,000/11,389	12,063/11,453	64/65	-4/13			H/H	67.69
*atp*6	12,064/11,454	12,579/11,966	516/513		ATG/ATG	TAA/TAG	H/H	69.56
*nad*2	12,629/11,962	13,525/12,810	897/849	49/-5	TTG/ATT	TAA/TAA	H/H	56.16
*trnA*	13,559/12,817	13,624/12,878	66/62	33/6			H/H	68.18
*trnD*	13,633/12,880	13,697/12,941	65/62	8/1			H/H	83.08
*trnV*	13,698/12,944	13,763/13,010	66/67	-/2			H/H	86.76
*nad*1	13,764/13,012	14,660/13,905	897/894	-/1	GTG/ATG	TAG/TAA	H/H	69.56
*trnN*	14,661/13,909	14,726/13,971	66/63	-/3			H/H	74.63
*trnI*	14,759/14,056	14,827/14,123	69/68	32/11			H/H	77.14
*trnP*	14,854/13,978	14,920/14,044	67/67	26/6			H/H	79.41
*trnK*	14,923/14,130	14,986/14,197	64/68	2/6			H/H	69.12
*nad*3	15,018/14,200	15,386/14,550	369/351	31/2	ATG/ATG	TAG/TAG	H/H	63.44
*trnW*	15,367/14,549	15,433/14,614	67/66	-20/-2			H/H	66.18

*Abbreviation*: LNCR, large non-coding region

### Protein-coding genes and codon usage

Eleven out of 12 PCGs of the two studied mitogenomes used ATG or GTG as the initial codons. However, it proved difficult to determine the initial codon of the *nad*2 gene in both species. On the basis of results reported for other related species, as a working hypothesis we proposed TTG and ATT as the initial codon of *nad*2 for *L. longipenis* and *L. spari*, respectively. Similarly, ATT was proposed as the start codon for *nad*2 in *B. hoshinai* (Ogawa, 1984) and *Aglaiogyrodactylus forficulatus* (Kritsky, Vianna & Boeger, 2007) [20, 50], and TTG as the start codon for *cox*2 in *Paragyrodactylus variegatus* (You, King, Ye & Cone, 2014) [51] (also see Additional file 3: Table S2). Canonical stop codons for the genetic code 9 (echinoderm and flatworm mitochondrion), TAA and TAG, were found in all 12 PCGs (Table 1 and Additional file 3: Table S2). Codon usage, RSCU, and codon family (corresponding to the amino acids) proportions were investigated among the seven available capsalids (three species) and dactylogyrids (four species) (Additional file 4: Figure S1). The third codon position exhibited the highest A+T bias (Table 2). Amino acids encoded by adenosine and thymine-rich codon families (such as Phe, Leu2 and Ile) were strongly preferred, whereas amino acids encoded by guanine and cytosine-rich codon families (such as Arg, Pro and Ala) appear to be selected against (Additional file 4: Figure S1).

**Table 2:** Nucleotide composition and skewness comparison of different elements of the mitochondrial genomes of *Lamellodiscus spari* and *Lepidotrema longipenis*

Regions	Size (bp)	T(U)	C	A	G	AT (%)	GC (%)	GT (%)	AT skew	GC skew
*Lepidotrema longipenis*/*Lamellodiscus spari*										
PCGs	9990/9780	47.3/47.7	7.9/8.5	28.5/26.7	16.3/17.1	75.8/74.4	24.2/25.6	63.6/64.8	-0.248/-0.281	0.347/0.336
1st codon position	3330/3260	41.2/40.3	8.2/9.0	30.4/29.5	20.2/21.3	71.6/69.8	28.4/30.3	61.4/61.6	-0.151/-0.154	0.425/0.407
2nd codon position	3330/3260	48.9/49.1	12.2/12.7	21.1/20.0	17.9/18.2	70.0/69.1	30.1/30.9	66.8/67.3	-0.398/-0.420	0.189/0.176
3rd codon position	3330/3260	51.7/53.7	3.4/3.8	34.0/30.7	10.8/11.8	85.7/84.4	14.2/15.6	62.5/65.5	-0.207/-0.273	0.523/0.516
*atp6*	516/513	49.0/51.9	8.7/9.2	25.0/23.6	17.2/15.4	74.0/75.5	25.9/24.6	66.2/67.3	-0.325/-0.375	0.328/0.254
*cox*1	1548/1557	44.4/45.6	10.2/10.6	26.0/25.3	19.4/18.5	70.4/70.9	29.6/29.1	63.8/64.1	-0.261/-0.286	0.310/0.272
*cox*2	576/582	42.0/43.5	9.7/8.9	29.3/26.1	18.9/21.5	71.3/69.6	28.6/30.4	60.9/65.0	-0.178/-0.249	0.321/0.412
*cox*3	654/651	52.1/50.4	6.0/7.2	26.6/25.3	15.3/17.1	78.7/75.7	21.3/24.3	67.4/67.5	-0.324/-0.331	0.439/0.405
*cytb*	1089/1089	45.7/46.2	9.6/11.1	27.4/24.9	17.4/17.8	73.1/71.1	27.0/28.9	63.1/64.0	-0.251/-0.300	0.290/0.232
*nad*1	897/894	46.9/47.9	6.4/7.8	28.2/26.1	18.5/18.2	75.1/74.0	24.9/26.0	65.4/66.1	-0.249/-0.295	0.489/0.399
*nad*2	897/849	51.3/50.8	7.4/6.9	30.2/28.5	11.1/13.8	81.5/79.3	18.5/20.7	62.4/64.6	-0.259/-0.281	0.205/0.330
*nad*3	369/351	48.5/45.9	4.6/5.1	31.2/33.0	15.7/16.0	79.7/78.9	20.3/21.1	64.2/61.9	-0.218/-0.162	0.547/0.514
*nad*4	1218/1176	48.1/48.7	8.9/8.6	28.7/26.7	14.4/16.0	76.8/75.4	23.3/24.6	62.5/64.7	-0.253/-0.292	0.237/0.301
*nad*4L	249/255	50.2/47.8	4.8/7.5	31.3/25.9	13.7/18.8	81.5/73.7	18.5/26.3	63.9/66.6	-0.232/-0.298	0.478/0.433
*nad*5	1527/1404	46.6/47.3	6.4/7.5	31.6/28.8	15.5/16.3	78.2/76.1	21.9/23.8	62.1/63.6	-0.192/-0.242	0.413/0.367
*nad*6	450/459	48.9/49.0	6.9/5.4	27.8/29.8	16.4/15.7	76.7/78.8	23.3/21.1	65.3/64.7	-0.275/-0.243	0.410/0.485
*rrnL*	973/952	41.7/40.1	8.6/9.5	34.0/34.3	15.6/16.1	75.7/74.4	24.2/25.6	57.3/56.2	-0.102/-0.078	0.288/0.259
*rrnS*	720/737	39.7/38.8	9.6/10.2	35.3/33.9	15.4/17.1	75.0/72.7	25.0/27.3	55.1/55.9	-0.059/-0.067	0.233/0.254
LNCR	1993/1773	46.3/40.3	2.6/2.8	47.5/52.2	3.6/4.7	93.8/92.5	6.2/7.5	49.9/45.0	0.013/0.128	0.161/0.263
tRNAs	1448/1237	40.2/40.4	7.9/8.4	37.0/34.2	14.9/17.0	77.2/74.6	22.8/25.4	55.1/57.4	-0.041/-0.083	0.309/0.338
rRNAs	1693/1689	40.9/39.6	9.0/9.8	34.6/34.2	15.5/16.5	75.5/73.8	24.5/26.3	56.4/56.1	-0.084/-0.073	0.264/0.257
Full genome	15,433/14,614	45.8/45.3	7.3/7.9	32.6/31.5	14.3/15.4	78.4/76.8	21.6/23.3	60.1/60.7	-0.169/-0.180	0.326/0.323

*Abbreviation*: LNCR, large non-coding region

### Loss of *trnS*1, *trnC* and *trnG* from the *L. spari* mitogenome

**Fig. 2:**
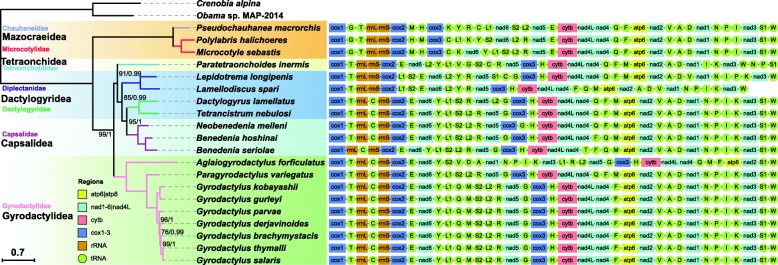
Phylogeny and mitogenomic architecture of the class Monogenea. The phylogram was constructed using MTART model on the basis of concatenated amino acid sequences of 20 monogenean mitogenomes. *Crenobia alpina* and *Obama* sp. are outgroups. The scale-bar corresponds to the estimated number of substitutions per site. Statistical support values are shown above the nodes, except for nodes with maximum support. Monogenean families are shown in different colors. Gene order is displayed to the right of the tree

Both ARWEN and MITOS algorithms failed to detect *trnS*1, *trnC* and *trnG* in the *L. spari* mitogenome. We made several attempts to corroborate that this is not an artifact. First, we carefully checked (*via* alignments with monogenean *trnS*1, *trnC* and *trnG* homologs) all intergenic sequences (including the LNCR). As these missing tRNA genes are located between *nad*5 and LNCR in *L. longipenis* (Table 1, Fig. 2), and as *trnC* is located between two rRNA genes in many other monogeneans (Fig. 2), we focused specifically on these two fragments. None of these sequences showed appreciable similarity with the queried homologs. Secondly, we re-sequenced the fragment between *nad*5 and *cox*3 using both mixture DNA and individual DNA, and checked the chromatograms carefully [52]. We did not find any evidence for sequence variability. Thirdly, referring to *L. longipenis* and other closely related monogeneans, as well as cestodes and trematodes in most of which *trnG* is located between *nad*5 and *cox*3 (Fig. 2, Additional file 5: Figure S2), we designed two primer pairs (Additional file 1: Dataset S1) to assess whether *trnG* is located between these two genes (or in their vicinity) in *L. spari*: (i) one forward primer (LS-GlyF) matching the most conserved region of *trnG* and two reverse primers (LSR1-6 and LSR1-8) matching the conserved regions of *cox*3 to amplify the fragment between *trnG* and *cox*3; and (ii) two forward primers (LSF17-0 and LSF17) matching *nad*5 and one reverse primer (LS-GlyR) matching *trnG* to amplify the fragment between *nad*5 and *trnG*. None of these primer pairs could generate a PCR product, which indicates that *trnG* is either not in the vicinity of these two genes in *L. spari*, or that its sequence is highly divergent, or that it is completely missing from the mitogenome. On the basis of these tests and high quality of chromatograms of the fragment between *nad*5 and *cox*3 [52], we suspect that all three tRNAs (*trnS*1*-trnC-trnG*) might be missing from the mitogenome of *L. spari*. These results would have to be corroborated either by resequencing of this mitogenome, or by sequencing of other closely related species. Loss of tRNA genes was also reported in many other metazoan taxa [53–57]. Given that the amino acid usage frequency of serine (AGN), glycine and cysteine is analogous between *L. spari* and other monogeneans (Additional file 4: Figure S1), there are at least three possible explanations for the missing tRNAs: (i) they are imported from the nucleus, as is common in mitochondria [58]; (ii) they are encoded in the mitogenome, but undergo extensive post-transcriptional RNA editing [54], so they could not be identified from their coding sequences; and (iii) they are encoded on a separate minicircle of mtDNA.

### Non-coding regions

A putative control region, or long non-coding region (LNCR), was found between *nad*5 and *cox*3 genes in both mitogenomes (disregarding the three missing tRNAs; Table 1 and Fig. 1). The LNCR of *L. longipenis* (1993 bp) was the longest among the monogeneans characterized so far [4, 7, 16, 19, 20, 50, 51, 59–67]. The A+T content of both LNCRs (*L. longipenis* = 93.8%, *L. spari* = 92.5%) was much higher than in other parts of the mitogenomes (Table 2). Both LNCRs contained a highly repetitive region (HRR): the HRR of *L. longipenis* was composed of 18 tandem repeats (TRs), where repeat units 2–18 were identical (91 bp) and unit 1 was 1 bp longer with a nucleotide insertion at the 16th position; the HRR of *L. spari* contained 20 TRs with the consensus size of 87 bp, but sizes and sequences of the repeat units were variable, exhibiting nucleotide mutations, deletions and insertions. This is the second report of TRs with high repeat numbers and large size in the subclass Monopisthocotylea; the first report was also in a dactylogyridean species, *D. lamellatus* [19]. These findings consistently reject the hypothesis that monopisthocotylids possess fewer and smaller (in size) TRs in the LNCR than polyopisthocotylids [61]. As in other monogeneans [19, 64] and cestodes [68, 69], both consensus repeat patterns of the HRRs in *L. longipenis* and *L. spari* are capable of forming stem-loop structures (Additional file 6: Figure S3). Since the presence of tandem repeats forming stable secondary structure is often associated with replication origin in mitochondria [64, 70, 71], it appears likely that these repeat regions are embedded within the control region.

**Fig. 3:**
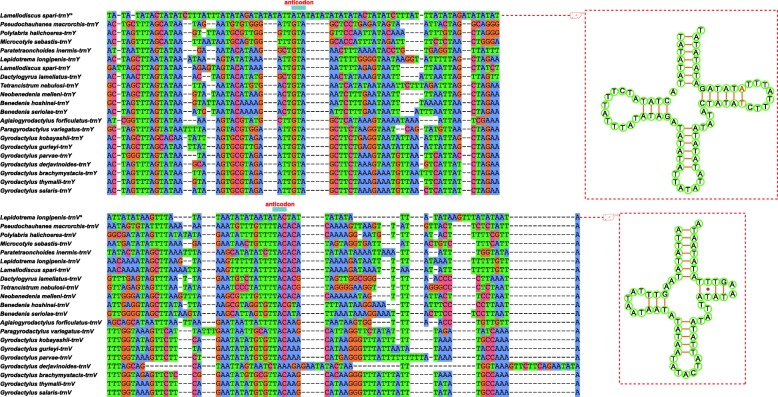
Alignment of *trnV* and *trnY* pseudogenes with the corresponding functional monogenean tRNA homologs. Cloverleaf structures of the two pseudogenes are shown on the right of alignment. Two red bases in *trnY* indicate nucleotide insertions in the TΨC stem

Aside from these TRs, the LNCR of *L. longipenis* also harbored 17 identical *trnV* pseudogenes, whereas the LNCR of *L. spari* contained 13 *trnY* pseudogenes (identified using ARWEN and DOGMA algorithms), all of which were located on the minus strand. These pseudogenes and HRR repeat patterns were two separate features, although the pseudogenes partially overlapped with the repeat patterns of HRR. Among the 13 *trnY* pseudogenes, six were 80 bases-long, and seven were 82 bases-long, with two bases inserted in the TΨC stem. *trnV* pseudogene had a standard TAC anticodon, whereas *trnY* contained modified standard anticodon (ATA). The cloverleaf structures of the two pseudogenes and the alignment with the corresponding functional monogenean tRNA homologs is shown in Fig. 3. Average sequence similarity values of the alignment for *trnV* and *trnY* were 40.78 ± 3.87% and 39.91 ± 4.13%, respectively. As the amino acid usage frequencies of valine and tyrosine for *L. longipenis* and *L. spari* were analogous with other monogeneans (Additional file 4: Figure S1), we hypothesise that the presence of these pseudogenes is non-adaptive, and that they may not be functional. A tRNA pseudogene was also found in the LNCR of *Paratetraonchoides inermis* (Bychowsky, Gussev & Nagibina, 1965) [4], but such a large accumulation of tRNA pseudogenes in tandem arrays is much more common in plastid genomes [72] and prokaryotes [73] than in mitochondrial genomes of metazoans. It might be of interest to sequence mitogenomes of other closely related species to infer whether they also harbor this feature, and study its evolutionary history and mutational rate.

### Nucleotide diversity and evolutionary rate analysis

**Fig. 4:**
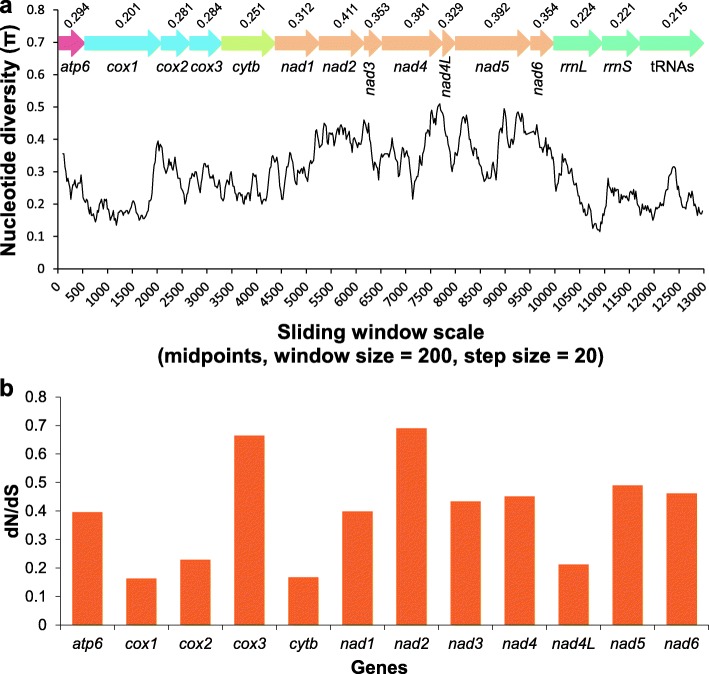
Sliding window and selection pressure analyses of the mitogenomes of *Lepidotrema longipenis* and *Lamellodiscus spari*. **a** Sliding window analysis was conducted on concatenated alignments of 12 PCGs, 2 rRNAs and 19 coalescent tRNAs (missing tRNAs, *trnS*1, *trnC* and *trnG*, were removed). The black line represents the value of nucleotide diversity (window size = 200 bp, step size = 20 bp, with the value inserted at its mid-point). Gene names, boundaries/direction (colored arrows) and average nucleotide diversity values are indicated above the graph. **b** Ratios of non-synonymous (dN) to synonymous (dS) substitution rates calculated for protein-coding genes

The sliding window analysis was conducted using concatenated alignments of 12 PCGs, two rRNAs and 19 coalescent tRNAs of the two diplectanids (*trnS*1, *trnC* and *trnG* were removed due to their absence from *L. spari*). The plot of sequence variation ratio exhibited highly variable nucleotide diversity between the two diplectanids, with Pi values for the 200 bp windows ranging from 0.201 to 0.411 (Fig. 4a). *cox*1 (0.201), tRNAs (0.215), *rrnS* (0.221), *rrnL* (0.224) and *cytb* (0.251) exhibited a comparatively low sequence variability, whereas *nad*2 (0.411), *nad*5 (0.392), *nad*4 (0.381) and *nad*6 (0.354) had a comparatively high sequence variability. This was corroborated by the non-synonymous/synonymous (dN/dS) ratio (omega) analysis, which showed that *cox*1 (0.163), *cytb* (0.167), *nad*4L (0.213) and *cox*2 (0.229) are evolving comparatively slowly, whereas *nad*2 (0.69), *nad*5 (0.49), *nad*6 (0.462) and *nad*4 (0.451) are evolving comparatively fast (Fig. 4b). Therefore, these analyses consistently indicate that *cox*1, which is often used as a universal barcode for species identification [74], as well as population genetics in monogeneans [75–78], is the slowest evolving and least variable gene. As rapidly evolving genes are more suitable for analyzing relationships among closely related species [79], we propose that the fast-evolving *nad*2, *nad*5, *nad*6 and *nad*4 would be better molecular markers than *cox*1 for diplectanids.

### Phylogeny

Regardless of the dataset and model used, all analyses produced phylograms with concordant branch topologies (Fig. 2 and Additional file 7: Figure S4 and S5). As the AliGROOVE analysis indicated that the PCGAA dataset exhibits lower heterogeneity than PCGRT (Additional file 7: Figure S6), we displayed only the results of PCGAA in Fig. 2. As expected, the two diplectanids, *L. longipenis* and *L. spari*, constituted a monophyletic group with maximum support. In accordance with previous results based on mitochondrial phylogenomics [4, 19, 20], the two dactylogyrids (*D. lamellatus* and *T. nebulosi*; Dactylogyridea: Dactylogyridae) formed a sister-group with the three capsalids (*N. melleni*, *B. hoshinai* and *B. seriolae*; Capsalidea: Capsalidae). The two newly-sequenced diplectanids (Diplectanidae) formed a sister-group with this (Dactylogyridae + Capsalidae) clade. As the family Diplectanidae was classified into the order Dactylogyridea in the classifications proposed by Bychowsky [80], Lebedev [81] and Boeger & Kritsky [82], this topology rendered Dactylogyridea paraphyletic by the nested Capsalidea clade. In these taxonomic systems, the Dactylogyridae and Diplectanidae were assigned to the same order Dactylogyridea (or suborder Dactylogyrinea within the order Dactylogyridea in Bychowsky [80]), whereas the Capsalidae was classified into the order Capsalidea (or suborder Monopisthocotylinea within the order Dactylogyridea in Bychowsky [80]). A number of subsequent morphology- and molecular data-based studies further supported the closer phylogenetic relationship between the Dactylogyridae and Diplectanidae than either of the two with Capsalidae: chaetotaxy and ciliated cells of the oncomiracidium [83], spermatozoon ultrastructure [12, 13], comprehensive morphological characters [14, 82], *28S* rRNA [8] and *18S* rRNA [9–11]. Regarding the phylogenetic position of the Capsalidae, it was either resolved as phylogenetically closely related to the Gyrodactylidea (families Gyrodactylidae and/or Udonellidae) based on the evidence of spermatozoon ultrastructure [12, 13], *28S* rRNA gene [8], *18S* rRNA gene [9–11], and a combination of three unlinked nuclear genes (*28S* rRNA, *Histone 3* and *Elongation Factor 1α*) [5], or was resolved as basal to the Gyrodactylidea and Dactylogyridea on the basis of comprehensive morphological characters [14, 82]. However, as argued before, morphological traits are liable to cause taxonomic and phylogenetic artifacts in (parasitic) microscopic animals [4–6] and single gene-based molecular markers may not provide sufficient resolution power to infer the relationships among these species with high precision [7, 84]. These discrepancies could also be a result of discrepant evolutionary rates between mitochondrial and nuclear sequences, which can produce differing evolutionary signals [85, 86]. Although our phylogenetic analysis was based on relatively limited mitogenomic data (four species belonging to two families for Dactylogyridea, and three species/one family for Capsalidea), the paraphyly of the Dactylogyridea with reasonably high support values (BS = 85, PB = 0.99) suggests that their relationships should be further explored using a larger number of monogenean mitogenomes and large nuclear datasets.

### Gene order

The order of tRNA genes of the two studied diplectanids exhibits notable rearrangements in comparison to all other sequenced monogenean mitogenomes (Fig. 2, Additional file 8: Table S4). Disregarding the three missing tRNAs, the gene order of the two diplectanids was very similar, with only two transposition events: position interchanges of *trnF* and *trnQ*, and *trnP* and *trnI* (Fig. 2 and Additional file 8: Table S4). This unique gene order pattern of the two diplectanids is further manifested by low pairwise similarity values in comparison with those observed among other monogeneans: the highest, between *L. spari* and *B. hoshinai*, is only 354 over 1254 (Additional file 8: Table S4). The transformational pathway from *L. spari* to the most similar gene arrangement, belonging to *B. hoshinai*, required one transposition, one TDRL (tandem-duplication-random-loss) and two coupled transposition events (Additional file 9: Figure S7). The transformational pathway from *L. longipenis* to the most similar gene arrangement found in *T. nebulosi* and *P. variegatus* (similarity value: 322 over 1254; gene orders of *T. nebulosi* and *P. variegatus* were identical) required two transpositions, one TDRL and two coupled transposition events (Additional file 9: Figure S7). In addition to the patterns summarized in our recent paper [4], we assigned pattern 1c to the gene order of the two diplectanids, which seems to be synapomorphic to the family Diplectanidae (Additional file 5: Figure S2).

Conserved gene arrangement is considered to be a typical feature of mitochondrial genomes [87–89], but our results suggest that extensive gene order rearrangements are not rare events in the class Monogenea. As five out of six proposed main patterns are found in the Monogenea (1a, 1b, 3, 4 and the new pattern 1c; Additional file 5: Figure S2), this further confirms the hypothesis [4] that gene order in monogeneans is evolving at a relatively rapid rate. However, evidence is emerging that the evolution of mitogenomic gene order arrangements is discontinuous in monogeneans, as some taxonomic categories appear to be particularly prone to mitogenomic rearrangements (diplectanids, tetraonchids and *A. forficulatus*), whereas others exhibit relatively conserved gene orders. Of course, our conclusions should be interpreted within the context of the limited number of monogenean mitogenomes currently available. Discontinuity in mitogenomic architecture evolution was also found in nematodes [28], snails [90], insects [91] and vertebrates [92]. Although gene order is sometimes used as a tool for inferring phylogenetic relationships [93, 94], this discontinuity in gene order rearrangements in monogeneans might produce misleading evolutionary signals, such as disproportionately long branches, which in turn might cause long branch attraction artifacts. Thus, gene order may only be used for phylogenetic analyses in this group of animals with this limitation in mind. The provisional addition of three missing tRNAs certainly affected the similarity values and transformational pathways between *L. spari* and other monogeneans, but it would not affect the assignment of a new gene order pattern to diplectanids, nor our conclusion that diplectanids are prone to mitogenomic rearrangements.

## Conclusions

The present study reports four findings worthy of emphasis. First, on the basis of nucleotide diversity, dN/dS and average sequence identity, we propose that *nad*2, *nad*5 and *nad*4 genes are better-suited as molecular markers for species identification and population genetics studies of diplectanids than the commonly used *cox*1. Secondly, the long non-coding region of both mitogenomes contains two interesting features: (i) a highly repetitive region, which is often associated with replication origin in mitochondria; and (ii) tRNA pseudogenes in tandem arrays, which is common in plastid genomes and prokaryotes, but rare in metazoan mitogenomes. Thirdly, phylogenetic analysis showed that the two new diplectanids (Dactylogyridea) formed a sister group with a clade comprised of two other dactylogyrids (Dactylogyridea) and three capsalids (Capsalidea). Thus, Dactylogyridea was rendered paraphyletic by the nested Capsalidea clade. Fourthly, due to the extensive tRNA gene rearrangements in the two diplectanids, we assigned them a new gene order pattern, and concluded that the evolution of mitogenomic gene order arrangements is discontinuous in monogeneans. However, our confidence in the unorthodox phylogeny produced here, and understanding of genomic architecture evolution, is curbed by the scarcity of available mitogenomes (only four dactylogyrideans and 20 monogeneans), so we encourage researchers to accumulate more samples and molecular data (especially large molecular data) for monogenean parasites in order to infer their evolutionary history with confidence.

## Additional files

Additional file 1: **Dataset S1.** Supplementary methods. (DOCX 41 kb)

Additional file 2: **Table S1.** Monogenean species and outgroups used for comparative mitogenomic and phylogenetic analyses. (XLSX 14 kb)

Additional file 3: **Table S2.** General statistics for the protein-coding and rRNA genes of the two diplectanids and 18 other available monogeneans. (XLSX 16 kb)

Additional file 4: **Figure S1.** Relative synonymous codon usage (RSCU) of six monopisthocotylid mitogenomes. (PDF 57 kb)

Additional file 5: **Figure S2.** The 23 unique gene orders in neodermatan mitochondrial genomes filtered from 113 species. Representative species and corresponding taxonomic categories at the class/subclass level are shown on the left; a star symbol denotes that the gene order is shared by Monogenea and Cestoda. Pattern types used here to classify gene orders are shown on the right. In *Lamellodiscus spari*, the missing tRNAs are represented by the “?” symbol in the positions homologous to the closest available diplectanid relative, *Lepidotrema longipenis*. (PDF 1961 kb)

Additional file 6: **Figure S3.** Stem-loop structures of the consensus repeat pattern in highly repetitive regions of the long non-coding regions of *Lepidotrema longipenis* and *Lamellodiscus spari*. dG denotes the structure’s free energy (ΔG in Kcal/mol at 37 °C). (PDF 297 kb)

Additional file 7: **Figure S4.** Maximum likelihood tree of nucleotide dataset with partition model. **Figure S5.** Maximum likelihood tree of amino acid dataset with partition model. **Figure S6.** AliGROOVE analysis. **Table S3.** The best partitioning scheme. (DOCX 517 kb)

Additional file 8: **Table S4.** Pairwise comparison of mitochondrial DNA gene orders among 13 monogenean species based on the order of all 36 genes. Scores correspond to the similarity between gene orders, where “1254” represents an identical gene order. N is the number identifying the taxon in the pairwise comparisons. (XLSX 11 kb)

Additional file 9: **Figure S7.** Transformational pathway from the gene orders of the two diplectanids to the most similar monogenean gene arrangements. (PDF 992 kb)

## Abbreviations

ORF: open reading frame
RSCU: relative synonymous codon usage
PCGs: protein-encoding genes
ML: maximum likelihood
BI: Bayesian inference
dN: non-synonymous mutation rate
dS: synonymous mutation rate
LNCR: long non-coding regions
BS: bootstrap
PB: PhyloBayes
HRR: highly repetitive region
TRs: tandem repeats
TDRL: tandem-duplication-random-loss
