# Supplementary material for: Mitochondrial genomes of two diplectanids (Platyhelminthes: Monogenea) expose paraphyly of the order Dactylogyridea and extensive tRNA gene rearrangements
Source: Parasit Vectors. 2018 Nov 20;11:601. doi: 10.1186/s13071-018-3144-6 (PMC6245931; doi:10.1186/s13071-018-3144-6)
Supplement: Supplementary file 1 — Dataset S1. Supplementary methods. (DOCX 41 kb) [file 13071_2018_3144_MOESM1_ESM.docx]

**Primers**

Primers used to verify the loss of *trnS1*, *trnC* and *trnG* in *Lamellodiscus spari*.

| Name | Sequence (5' to 3') |
| --- | --- |
| LS-GlyF | CTAWCTTTCCAARTTAGWGA |
| LSR1-6 | CCTAAAACTAAAGTTAAACCC |
| LSR1-8 | GCATCTAAATAATGCTTAAAAG |
| LSF17-0 | GGTTTAGTTAGAGGTTTAAATAGG |
| LSF17 | GTAGTTCATTAAATGAATTAACGG |
| LS-GlyR | TCWCTAAYTTGGAAAGWTAG |

Universal primers used to amplify the *28S rRNA* gene

| Name | Sequence (5' to 3') |
| --- | --- |
| C1 | ACCCGCTGAATTTAAGCAT |
| D2 | TGGTCCGTGTTTCAAGAC |

Primers used to verify the obtained sequences using individual DNA and long-range PCR

| **Primer name** | **Sequence (5’-3’)** | **Length (bp)** |
| --- | --- | --- |
| *Lamellodiscus spari* | |  |
| LSF1 | CAAGTTATACAAAGGTTGGTTTG | 4423 |
| LSR1 | CTTCCTCAACCAACTAGAAG |  |
| LSYZF2 | AGGACGAAGAAATACATCAC | 3910 |
| LSYZR2 | CGGTCTTAACTCAACTCATG |  |
| LSF4 | GTGAATAATGGAAAAGTTACC | 3716 |
| LSYZR3 | ACCACCTAAAAATGGCATC |  |
| LSF6 | GTAATTTTGGTTTAGTTAGAGG | 3235 |
| LSYZR4 | CCCACATAGGGAACTCTTTG |  |
| *Lepidotrema longipenis* | |  |
| LPCF1 | CTATACCAGGGCGAATCAATC | 7543 |
| LPCR1 | CCAACTCCTTAATAGCTGAAC |  |
| LPCYZF2 | GTTTCTATGGCACTTAGGG | 3381 |
| LPCYZR2 | CGTGGTAAAGTGCCTCGAAC |  |
| LPCF3 | TATGCTGAATCAGAGAGAG | 1929 |
| LPCYZR3 | GAGCCCATACTACACAACC |  |
| LPCF4 | TGGTCATCCAGAGGTATATG | 3257 |
| LPCYZR4 | GTTACAAGCACTCAATTATC |  |

Primers used to amplify the complete mitochondrial genomes

| **Fragment No.** | **Gene or region** | **Primer name** | **Sequence (5’-3’)** | **Length (bp)** |
| --- | --- | --- | --- | --- |
|  |  |  |  |  |
| *Lepidotrema longipenis* | |  |  |  |
| F1 | *COX2-ND4* | LPCF1 | CTATACCAGGGCGAATCAATC | 7543 |
|  |  | LPCR1 | CCAACTCCTTAATAGCTGAAC |  |
| F2 | *ND4* | MSCND4F | WGGTATGTTATGAAGTTNGG | 163 |
|  |  | MSCND4R | CTAAGAWAAGCAAGHCAACGC |  |
| F3 | *ND4-ND1* | LPCF2 | GTTATGAAGTTGGGTTTAATAGG | 2907 |
|  |  | LPCR2 | CTATATCTACTAATCCTAG |  |
| F4 | *ND1* | JQXCND1F | CGAAAGGNCCBTAANAAGGTTGG | 701 |
|  |  | JQXCND1R1 | CGAACHCGTGGTARAGTGCCTCG |  |
| F5 | *ND1-COX1* | LPCF3 | TATGCTGAATCAGAGAGAG | 1614 |
|  |  | LPCR3 | ACTACAGATATCATCTCATG |  |
| F6 | *COX1* | TXCCOX1F | GGTTGAACTHTTWATCCTCC | 455 |
|  |  | TXCCOX1R | GCAARAACTAAACCDAAAMAACC |  |
| F7 | COX1-12S | LPCF4 | TGGTCATCCAGAGGTATATG | 2234 |
|  |  | LPCR4 | CCCTAAATTAAACAAGTATC |  |
| F8 | *12S* | MSC12SF | CAGTGCCAGCATCTGCGGTT | 494 |
|  |  | MSC12SR | GATTGACGGGCGGTGTGTACCC |  |
| F9 | 12S-COX2 | LPCF5 | GCCAGGTCTATGTGCTACTG | 590 |
|  |  | LPCR5 | AACACTAAGGGATTATCTAC |  |
| F10 | COX2 | LPCF6 | AGTAGACGACACTAGTAAG | 328 |
|  |  | LPCR6 | TACTTCTACAACAATAGGC |  |
| *Lamellodiscus spari* | |  |  |  |
| F1 | *CYTB* | MSCCYTBF | CATATGGGWCGNGCATTGTA | 186 |
|  |  | MSCCYTBR | TTTACCCACATAGGGAACTC |  |
| F2 | CYTB-ND1 | LSF1 | CAAGTTATACAAAGGTTGGTTTG | 4423 |
|  |  | LSR1 | CTTCCTCAACCAACTAGAAG |  |
| F3 | *ND1* | MSCND1F | CGDAAGGGACCTAAAAAGGTTGG | 700 |
|  |  | MSCND1R | GAACACGTGGTAAAACACCACG |  |
| F4 | *ND1-COX1* | LSF2 | GAGCTGGTTTTTGTCAGTTG | 1446 |
|  |  | LSR2 | CTGACATAAATTCATGATTAATC |  |
| F5 | *COX1* | MSCCOX1F | GGDTGAACCTTYTATCCACC | 953 |
|  |  | MSCCOX1R | CADACTCGNCGTGGTAAACCAC |  |
| F6 | *COX1-16S* | LSF3 | GATACTTGATTTGTTGTTGCTC | 822 |
|  |  | LSR3 | GACACTTTACTAATTTGGTTAC |  |
| F7 | *16S* | MSC16SF | TTTACCTTTTGTATCATGAT | 841 |
|  |  | MSC16SR | NCGGTCTTAACTCAACTCAT |  |
| F8 | *16S-COX2* | LSF4 | GTGAATAATGGAAAAGTTACC | 1426 |
|  |  | LSR4 | CCTTTACACCAAGATCTGGTAC |  |
| F9 | COX2 | DZCOX2F1 | TCATDGGTCGTCAGTGGTATTG | 271 |
|  |  | DZCOX2R | CCBCACARTTCTCTACAATAACC |  |
| F10 | COX2-ND5 | LSF5 | GGTTGATGCAATCCCTGGTC | 2239 |
|  |  | LSR5 | CCGTTAATTCATTTAATGAACTAC |  |
| F11 | ND5-COX3 | LSF6 | GTAATTTTGGTTTAGTTAGAGG | 2322 |
|  |  | LSR6 | GACATAACTTAAAGTCTTCAG |  |
| F12 | COX3-CYTB | LSF7 | CTTTTAAGCATTATTTAGATGC | 1022 |
|  |  | LSR7 | CCACATAGGGAACTCTTTG |  |

**PCR conditions for degenerate primers:**

MSCCYTBF, MSCCYTBR, MSCND1F, MSCND1R, MSCCOX1F, MSCCOX1R, MSC16SF, MSC16SR, DZCOX2F1, DZCOX2R, MSCND4F, MSCND4R, JQXCND1F, JQXCND1R1, TXCCOX1F, TXCCOX1R, MSC12SF and MSC12SR. These primers were designed referring to 14 published monogenean mitogenomes (GenBank numbers: HM222526, KR871673, KY856918, AB731758, JQ038228, EF055880, AB905201, KM067269, KU679421, NC_021145, HQ009761, AP017665, NC_036219 and KT198989). Reaction mixture volume: 20 µl

Components:

- 7.4 µl dd H_2_O
- 10 µl 2×PCR buffer (Mg^2+^, dNTP plus, Takara, Dalian, China)
- 0.6 µl of each primer
- 0.4 µl r*Taq* polymerase (250 U, Takara)
- 1 µl of DNA template

Amplification conditions:

- initial denaturation at 98°C for 2 min
- 40 cycles of:
- 98°C for 10 s
- 50°C for 15 s
- 68°C for 1 min/kb
- final extension at 68°C for 10 min

**PCR conditions for specific primers:**

LSF1, LSR1, LSF2, LSR2, LSF3, LSR3, LSF4, LSR4, LSF5, LSR5, LSF6, LSR6, LSF7, LSR7, LPCF1, LPCR1, LPCF2, LPCR2, LPCF3, LPCR3, LPCF4, LPCR4, LPCF5, LPCR5, LPCF6, LPCR6

Reaction mixture volume: 20 µl

Components:

- 7.4 µl dd H_2_O
- 10 µl 2×PCR buffer (Mg^2+^, dNTP plus, Takara, Dalian, China)
- 0.6 µl of each primer
- 0.4 µl r*Taq* polymerase (250 U, Takara)
- 1 µl of DNA template

Amplification conditions:

- initial denaturation at 98°C for 2 min
- 40 cycles of:
- 98°C for 10 s
- 48–52°C for 15 s
- 68°C for 1 min/kb
- final extension at 68°C for 10 min

* Footnote:

Primer annealing temperature:

| Primer name | Temperature (°C) |
| --- | --- |
| LSF1/LSR1 | 52 |
| LSF2/LSR2 | 52 |
| LSF3/LSR3 | 52 |
| LSF4/LSR4 | 52 |
| LSF5/LSR5 | 50 |
| LSF6/LSR6 | 50 |
| LSF7/LSR7 | 50 |
| LPCF1/LPCR1 | 50 |
| LPCF2/LPCR2 | 48 |
| LPCF3/LPCR3 | 50 |
| LPCF4/LPCR4 | 50 |
| LPCF5/LPCR5 | 50 |
| LPCF6/LPCR6 | 48 |

**Sequence annotation and analyses**

After quality-proofing of the obtained fragments, the two mitogenomic sequences were assembled manually using DNAstar v7.1 software [1]. Both mitogenomes were annotated roughly following the procedure described before [2-5]. First, raw mitogenomic sequences were imported into MITOS web servers [6] to determine the approximate boundaries of genes. Exact positions of protein-coding genes (PCGs) were found by searching for ORFs (employing genetic code 9, echinoderm and flatworm mitochondrion). All tRNAs were identified using ARWEN [7], DOGMA [8] and MITOS. The precise boundaries of *rrnL* and *rrnS* were determined via a comparison with homologs. MitoTool [9], an in-house GUI-based software, was used to parse and extract the information from genomes manually annotated in Word documents, as well as create GenBank submission files and organization tables for mitogenomes. Genomic statistics of the two diplectanids, as well as all available monogeneans, was also extracted by MitoTool. Nucleotide sequences of 36 genes and amino acid sequences of twelve protein-coding genes (PCGs) were extracted from GenBank files using MitoTool. PCGs were aligned in batches using MAFFT [10], wherein amino acid sequences using normal mode, nucleotide sequences using codon model. 22 tRNAs and 2 rRNAs were aligned with Q-INS-i algorithm (which takes secondary structure information into account) incorporated into MAFFT-with-extensions software [10]. Ambiguously aligned fragments were removed from PCGRT and PCGAA using Gblocks [11]. To maximize the amount of phylogenetic signal retained in the data, Gblocks was used with ’relaxed’ parameters: minimum number of sequences for a conserved/flank position (11/22), maximum number of contiguous non-conserved positions (8), minimum length of a block (10), allowed gap positions (with half).

**References**

1. Burland TG. DNASTAR’s Lasergene sequence analysis software. Bioinformatics methods and protocols. 1999:71-91.

2. Zhang D, Zou H, Wu SG, Li M, Jakovlić I, Zhang J, Chen R, et al. Sequencing of the complete mitochondrial genome of a fish-parasitic flatworm *Paratetraonchoides inermis* (Platyhelminthes: Monogenea): tRNA gene arrangement reshuffling and implications for phylogeny. Parasit Vectors. 2017;10(1):462.

3. Zhang D, Zou H, Wu SG, Li M, Jakovlic I, Zhang J, Chen R, et al. Sequencing, characterization and phylogenomics of the complete mitochondrial genome of *Dactylogyrus lamellatus* (Monogenea: Dactylogyridae). J Helminthol. 2017:1-12.

4. Li WX, Zhang D, Boyce k, Xi BW, Zou H, Wu SG, Li M, et al. The complete mitochondrial DNA of three monozoic tapeworms in the Caryophyllidea: a mitogenomic perspective on the phylogeny of eucestodes. Parasit Vectors. 2017.

5. Zou H, Jakovlic I, Chen R, Zhang D, Zhang J, Li WX, Wang GT. The complete mitochondrial genome of parasitic nematode *Camallanus cotti*: extreme discontinuity in the rate of mitogenomic architecture evolution within the Chromadorea class. BMC Genomics. 2017;18(1):840.

6. Bernt M, Donath A, Juhling F, Externbrink F, Florentz C, Fritzsch G, Putz J, et al. MITOS: improved de novo metazoan mitochondrial genome annotation. Mol Phylogenet Evol. 2013;69(2):313-9.

7. Laslett D, Canback B. ARWEN: a program to detect tRNA genes in metazoan mitochondrial nucleotide sequences. Bioinformatics. 2008;24(2):172-5.

8. Wyman SK, Jansen RK, Boore JL. Automatic annotation of organellar genomes with DOGMA. Bioinformatics. 2004;20(17):3252-5.

9. Zhang D. MitoTool software. 2016. <https://github.com/dongzhang0725/MitoTool>. Accessed 5 May 2018.

10. Katoh K, Standley DM. MAFFT multiple sequence alignment software version 7: improvements in performance and usability. Mol Biol Evol. 2013;30(4):772-80.

11. Talavera G, Castresana J. Improvement of phylogenies after removing divergent and ambiguously aligned blocks from protein sequence alignments. Syst Biol. 2007;56(4):564-77.
