# Supplementary material for: Mitochondrial genomes of two diplectanids (Platyhelminthes: Monogenea) expose paraphyly of the order Dactylogyridea and extensive tRNA gene rearrangements
Source: Parasit Vectors. 2018 Nov 20;11:601. doi: 10.1186/s13071-018-3144-6 (PMC6245931; doi:10.1186/s13071-018-3144-6)
Supplement: Supplementary file 4 — Figure S1. Relative synonymous codon usage (RSCU) of six monopisthocotylid mitogenomes. (PDF 57 kb) [file 13071_2018_3144_MOESM4_ESM.pdf]

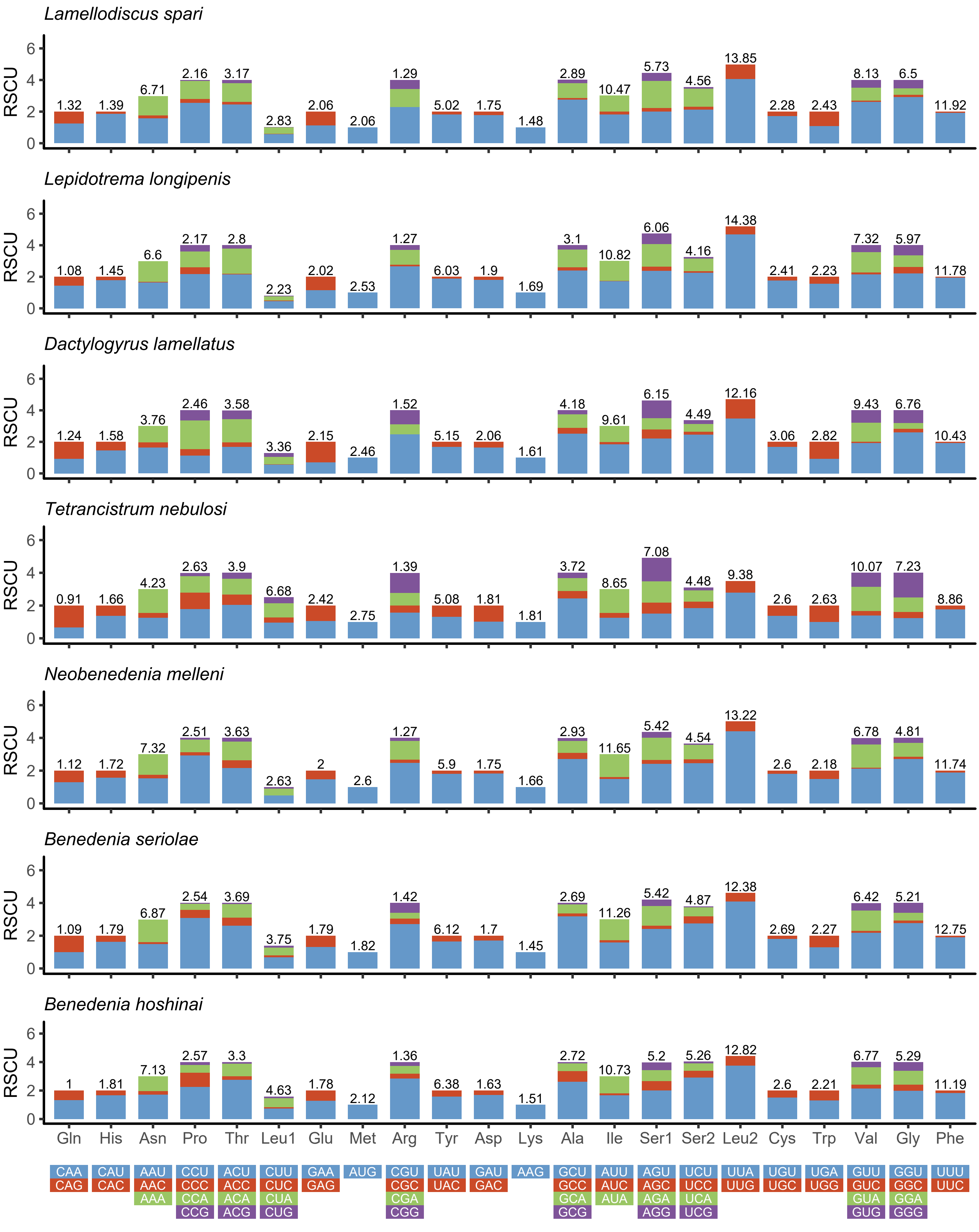

Lamellodiscus spari
RSCU
0 2 4 6
1.32 1.39 6.71 2.16 3.17 2.83 2.06 2.06 1.29 5.02 1.75 1.48 2.89 10.47 5.73 4.56 13.85 2.28 2.43 8.13 6.5 11.92
Lepidotrema longipenis
RSCU
0 2 4 6
1.08 1.45 6.6 2.17 2.8 2.23 2.02 2.53 1.27 6.03 1.9 1.69 3.1 10.82 6.06 4.16 14.38 2.41 2.23 7.32 5.97 11.78
Dactylogyrus lamellatus
RSCU
0 2 4 6
1.24 1.58 3.76 2.46 3.58 3.36 2.15 2.46 1.52 5.15 2.06 1.61 4.18 9.61 6.15 4.49 12.16 3.06 2.82 9.43 6.76 10.43
Tetrancistrum nebulosi
RSCU
0 2 4 6
0.91 1.66 4.23 2.63 3.9 6.68 2.42 2.75 1.39 5.08 1.81 1.81 3.72 8.65 7.08 4.48 9.38 2.6 2.63 10.07 7.23 8.86
Neobenedenia melleni
RSCU
0 2 4 6
1.12 1.72 7.32 2.51 3.63 2.63 2 2.6 1.27 5.9 1.75 1.66 2.93 11.65 5.42 4.54 13.22 2.6 2.18 6.78 4.81 11.74
Benedenia seriolae
RSCU
0 2 4 6
1.09 1.79 6.87 2.54 3.69 3.75 1.79 1.82 1.42 6.12 1.7 1.45 2.69 11.26 5.42 4.87 12.38 2.69 2.27 6.42 5.21 12.75
Benedenia hoshinai
RSCU
0 2 4 6
1 1.81 7.13 2.57 3.3 4.63 1.78 2.12 1.36 6.38 1.63 1.51 2.72 10.73 5.2 5.26 12.82 2.6 2.21 6.77 5.29 11.19
Gln His Asn Pro Thr Leu1 Glu Met Arg Tyr Asp Lys Ala Ile Ser1 Ser2 Leu2 Cys Trp Val Gly Phe
CAA CAU AAU CCU ACU CUU GAA AUG CGU UAU GAU AAG GCU AUU AGU UCU UUA UGU UGA GUU GGU UUU
CAG CAC AAC CCC ACC CUC GAG CGC UAC GAC GCC AUC AGC UCC UUG UGC UGG GUC GGC UUC
AAA CCA ACA CUA CGA GCA AUA AGA UCA GUA GGA
CCG ACG CUG CGG GCG AGG UCG GUG GGG
