# Supplementary material for: Mitochondrial genomes of two diplectanids (Platyhelminthes: Monogenea) expose paraphyly of the order Dactylogyridea and extensive tRNA gene rearrangements
Source: Parasit Vectors. 2018 Nov 20;11:601. doi: 10.1186/s13071-018-3144-6 (PMC6245931; doi:10.1186/s13071-018-3144-6)
Supplement: Supplementary file 5 — Figure S2. The 23 unique gene orders in neodermatan mitochondrial genomes filtered from 113 species. Representative species and corresponding taxonomic categories at the class/subclass level are shown on the left; a star symbol denotes that the gene order is shared by Monogenea and Cestoda. Pattern types used here to classify gene orders are shown on the right. In Lamellodiscus spari, the missing tRNAs are represented by the “?” symbol in the positions homologous to the closest available diplectanid relative, Lepidotrema longipenis. (PDF 1961 kb) [file 13071_2018_3144_MOESM5_ESM.pdf]

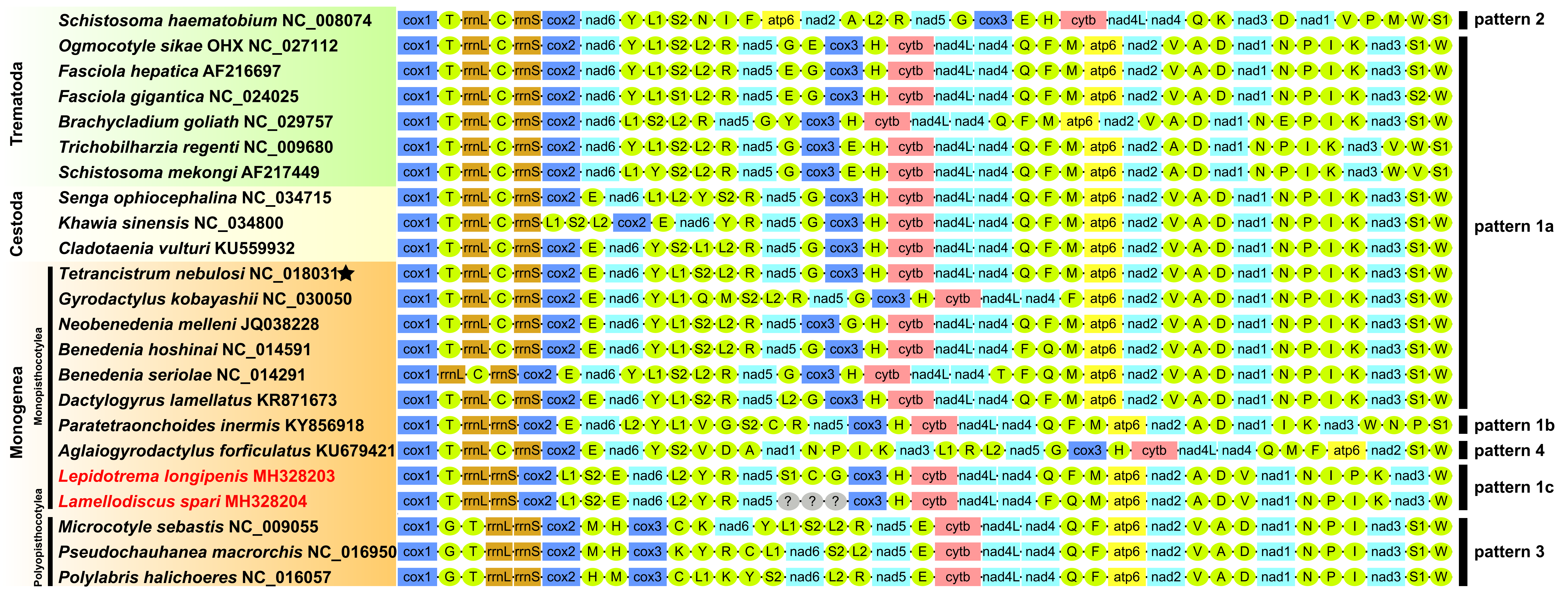
Trematoda
Cestoda
Monogenea
Monopisthocotylea
Polyopisthocotylea
Schistosoma haematobium NC_008074 cox1 T rrnL C rrnS cox2 nad6 Y L1 S2 N I F atp6 nad2 A L2 R nad5 G cox3 E H cytb nad4L nad4 Q K nad3 D nad1 V P M W S1
Ogmocotyle sikae OHX NC_027112 cox1 T rrnL C rrnS cox2 nad6 Y L1 S2 L2 R nad5 G E cox3 H cytb nad4L nad4 Q F M atp6 nad2 V A D nad1 N P I K nad3 S1 W
Fasciola hepatica AF216697 cox1 T rrnL C rrnS cox2 nad6 Y L1 S2 L2 R nad5 E G cox3 H cytb nad4L nad4 Q F M atp6 nad2 V A D nad1 N P I K nad3 S1 W
Fasciola gigantica NC_024025 cox1 T rrnL C rrnS cox2 nad6 Y L1 S1 L2 R nad5 E G cox3 H cytb nad4L nad4 Q F M atp6 nad2 V A D nad1 N P I K nad3 S2 W
Brachycladium goliath NC_029757 cox1 T rrnL C rrnS cox2 nad6 L1 S2 L2 R nad5 G Y cox3 H cytb nad4L nad4 Q F M atp6 nad2 V A D nad1 N E P I K nad3 S1 W
Trichobilharzia regenti NC_009680 cox1 T rrnL C rrnS cox2 nad6 Y L1 S2 L2 R nad5 G cox3 E H cytb nad4L nad4 Q F M atp6 nad2 A D nad1 N P I K nad3 V W S1
Schistosoma mekongi AF217449 cox1 T rrnL C rrnS cox2 nad6 L1 Y S2 L2 R nad5 G cox3 E H cytb nad4L nad4 Q F M atp6 nad2 A D nad1 N P I K nad3 W V S1
Senga ophiocephalina NC_034715 cox1 T rrnL C rrnS cox2 E nad6 L1 L2 Y S2 R nad5 G cox3 H cytb nad4L nad4 Q F M atp6 nad2 V A D nad1 N P I K nad3 S1 W
Khawia sinensis NC_034800 cox1 T rrnL C rrnS L1 S2 L2 cox2 E nad6 Y R nad5 G cox3 H cytb nad4L nad4 Q F M atp6 nad2 V A D nad1 N P I K nad3 S1 W
Cladotaenia vulturi KU559932 cox1 T rrnL C rrnS cox2 E nad6 Y S2 L1 L2 R nad5 G cox3 H cytb nad4L nad4 Q F M atp6 nad2 V A D nad1 N P I K nad3 S1 W
Tetrancistrum nebulosi NC_018031★ cox1 T rrnL C rrnS cox2 E nad6 Y L1 S2 L2 R nad5 G cox3 H cytb nad4L nad4 Q F M atp6 nad2 V A D nad1 N P I K nad3 S1 W
Gyrodactylus kobayashii NC_030050 cox1 T rrnL C rrnS cox2 E nad6 Y L1 Q M S2 L2 R nad5 G cox3 H cytb nad4L nad4 F atp6 nad2 V A D nad1 N P I K nad3 S1 W
Neobenedenia melleni JQ038228 cox1 T rrnL C rrnS cox2 E nad6 Y L1 S2 L2 R nad5 cox3 G H cytb nad4L nad4 Q F M atp6 nad2 V A D nad1 N P I K nad3 S1 W
Benedenia hoshinai NC_014591 cox1 T rrnL C rrnS cox2 E nad6 Y L1 S2 L2 R nad5 G cox3 H cytb nad4L nad4 F Q M atp6 nad2 V A D nad1 N P I K nad3 S1 W
Benedenia seriolae NC_014291 cox1 rrnL C rrnS cox2 E nad6 Y L1 S2 L2 R nad5 G cox3 H cytb nad4L nad4 T F Q M atp6 nad2 V A D nad1 N P I K nad3 S1 W
Dactylogyrus lamellatus KR871673 cox1 T rrnL C rrnS cox2 E nad6 Y L1 S2 R nad5 L2 G cox3 H cytb nad4L nad4 Q F M atp6 nad2 V A D nad1 N P I K nad3 S1 W
Paratetraonchoides inermis KY856918 cox1 T rrnL rrnS cox2 E nad6 L2 Y L1 V G S2 C R nad5 cox3 H cytb nad4L nad4 Q F M atp6 nad2 A D nad1 I K nad3 W N P S1
Aglaiogyrodactylus forficulatus KU679421 cox1 T rrnL C rrnS cox2 E nad6 Y S2 V D A nad1 N P I K nad3 L1 R L2 nad5 G cox3 H cytb nad4L nad4 Q M F atp6 nad2 S1 W
Lepidotrema longipenis MH328203 cox1 T rrnL rrnS cox2 L1 S2 E nad6 L2 Y R nad5 S1 C G cox3 H cytb nad4L nad4 Q F M atp6 nad2 A D V nad1 N I P K nad3 W
Lamellodiscus spari MH328204 cox1 T rrnL rrnS cox2 L1 S2 E nad6 L2 Y R nad5 ? ? ? cox3 H cytb nad4L nad4 F Q M atp6 nad2 A D V nad1 N P I K nad3 W
Microcotyle sebastis NC_009055 cox1 G T rrnL rrnS cox2 M H cox3 C K nad6 Y L1 S2 L2 R nad5 E cytb nad4L nad4 Q F atp6 nad2 V A D nad1 N P I nad3 S1 W
Pseudochauhanea macrorchis NC_016950 cox1 G T rrnL rrnS cox2 M H cox3 K Y R C L1 nad6 S2 L2 nad5 E cytb nad4L nad4 Q F atp6 nad2 V A D nad1 N P I nad3 S1 W
Polylabris halichoeres NC_016057 cox1 G T rrnL rrnS cox2 H M cox3 C L1 K Y S2 nad6 L2 R nad5 E cytb nad4L nad4 Q F atp6 nad2 V A D nad1 N P I nad3 S1 W
pattern 2
pattern 1a
pattern 1b
pattern 4
pattern 1c
pattern 3
