# Supplementary figures and images for: Mitochondrial genomes of two diplectanids (Platyhelminthes: Monogenea) expose paraphyly of the order Dactylogyridea and extensive tRNA gene rearrangements
Source: Parasit Vectors. 2018 Nov 20;11:601. doi: 10.1186/s13071-018-3144-6 (PMC6245931; doi:10.1186/s13071-018-3144-6)

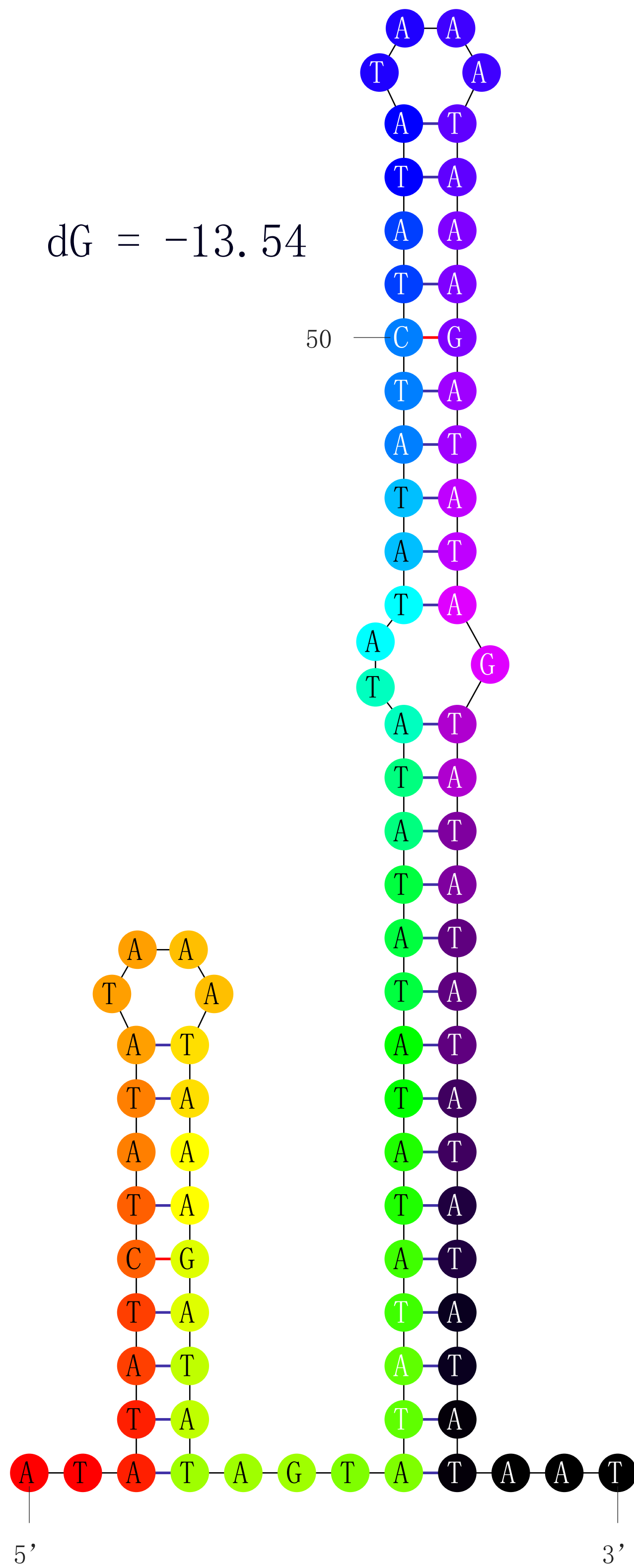

*Lamellodiscus spari*

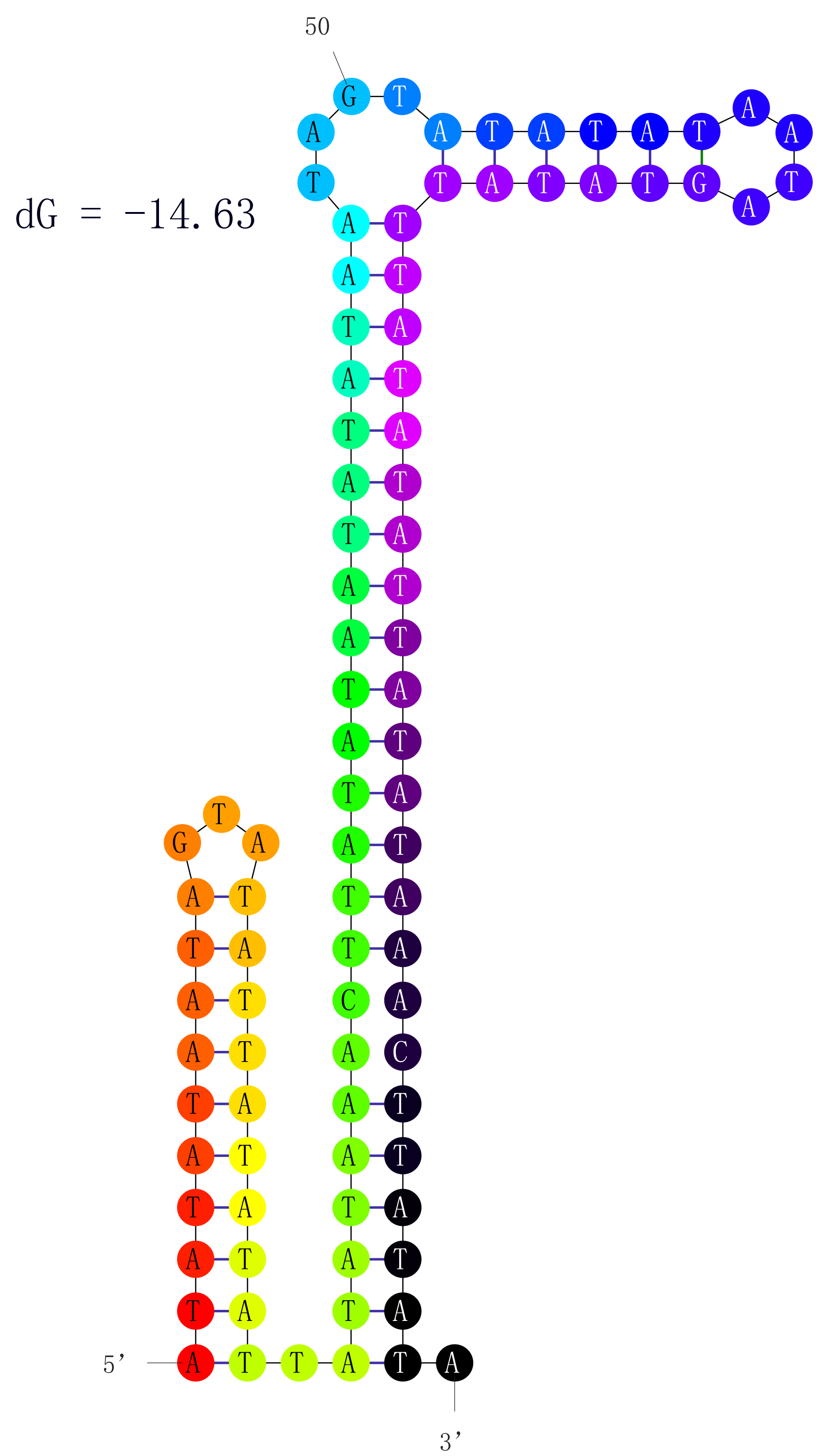

*Lepidotrema longipenis*

Supplement: Supplementary file 6 — Figure S3. Stem-loop structures of the consensus repeat pattern in highly repetitive regions of the long non-coding regions of Lepidotrema longipenis and Lamellodiscus spari. dG denotes the structure’s free energy (ΔG in Kcal/mol at 37 °C). (PDF 297 kb) [file 13071_2018_3144_MOESM6_ESM.pdf]

*Lamellodiscus spari* → *Benedenia hoshinai*

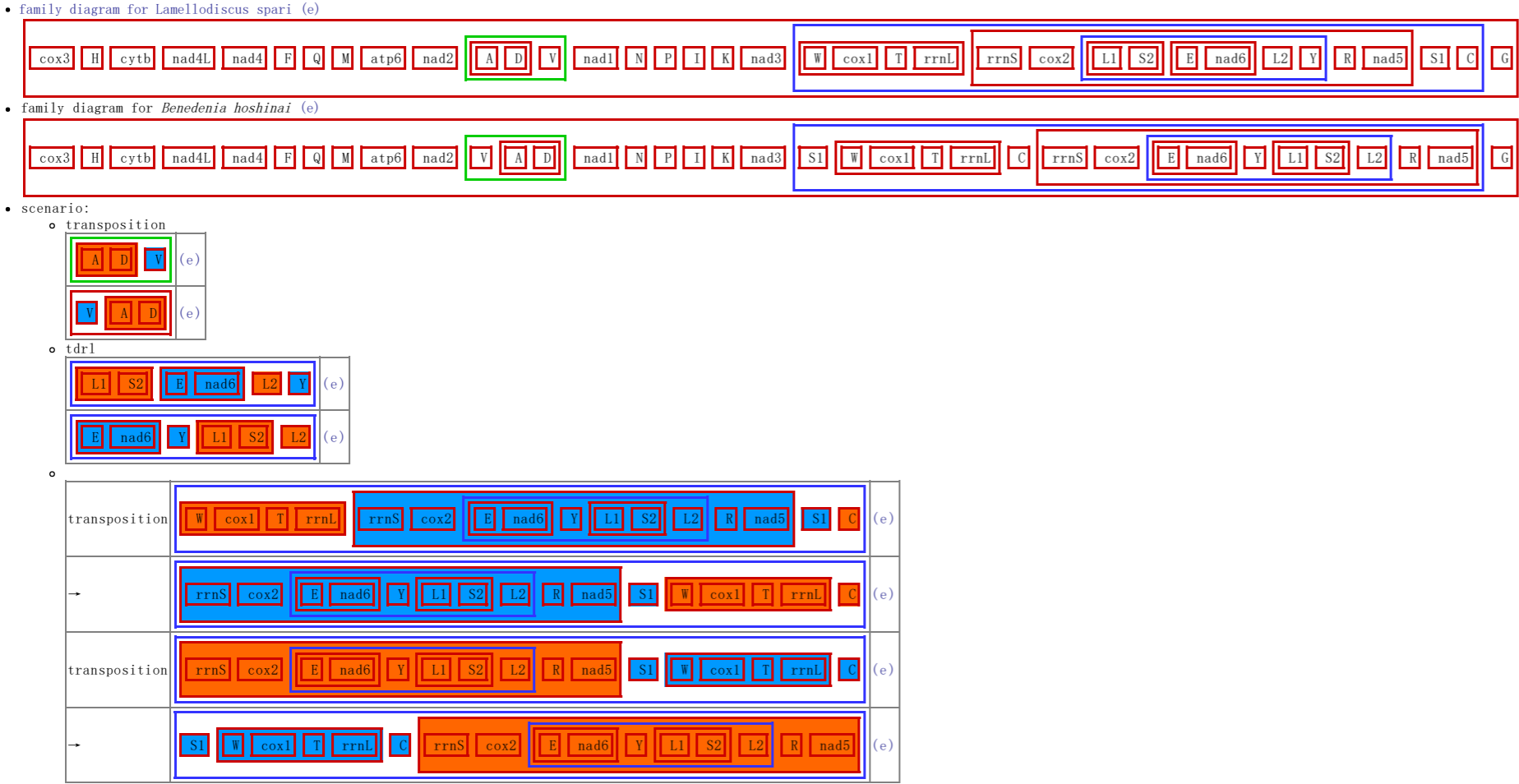

Supplement: Supplementary file 9 — Figure S7. Transformational pathway from the gene orders of the two diplectanids to the most similar monogenean gene arrangements. (PDF 992 kb) [file 13071_2018_3144_MOESM9_ESM.pdf]
