# Supplementary material for: Mitochondrial genomes of two diplectanids (Platyhelminthes: Monogenea) expose paraphyly of the order Dactylogyridea and extensive tRNA gene rearrangements
Source: Parasit Vectors. 2018 Nov 20;11:601. doi: 10.1186/s13071-018-3144-6 (PMC6245931; doi:10.1186/s13071-018-3144-6)
Supplement: Supplementary file 7 — Figure S4. Maximum likelihood tree of nucleotide dataset with partition model. Figure S5. Maximum likelihood tree of amino acid dataset with partition model. Figure S6. AliGROOVE analysis. Table S3. The best partitioning scheme. (DOCX 517 kb) [file 13071_2018_3144_MOESM7_ESM.docx]

**Additional file 7. Homogeneity and phylogenetic analyses**

Figure S4 Maximum likelihood phylogram constructed using PCGRT dataset with partition model (see Table S1 for partitioning scheme).


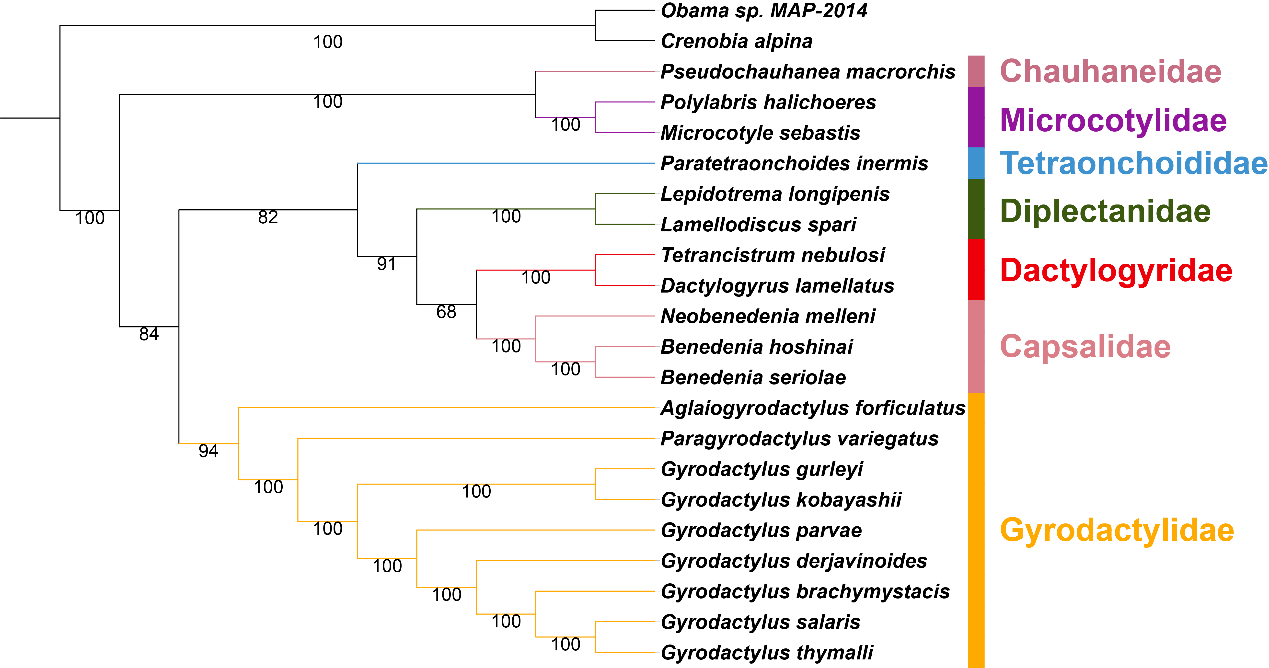


Figure S5 Maximum likelihood phylogram constructed using PCGAA dataset with partition model (see Table S1 for partitioning scheme).


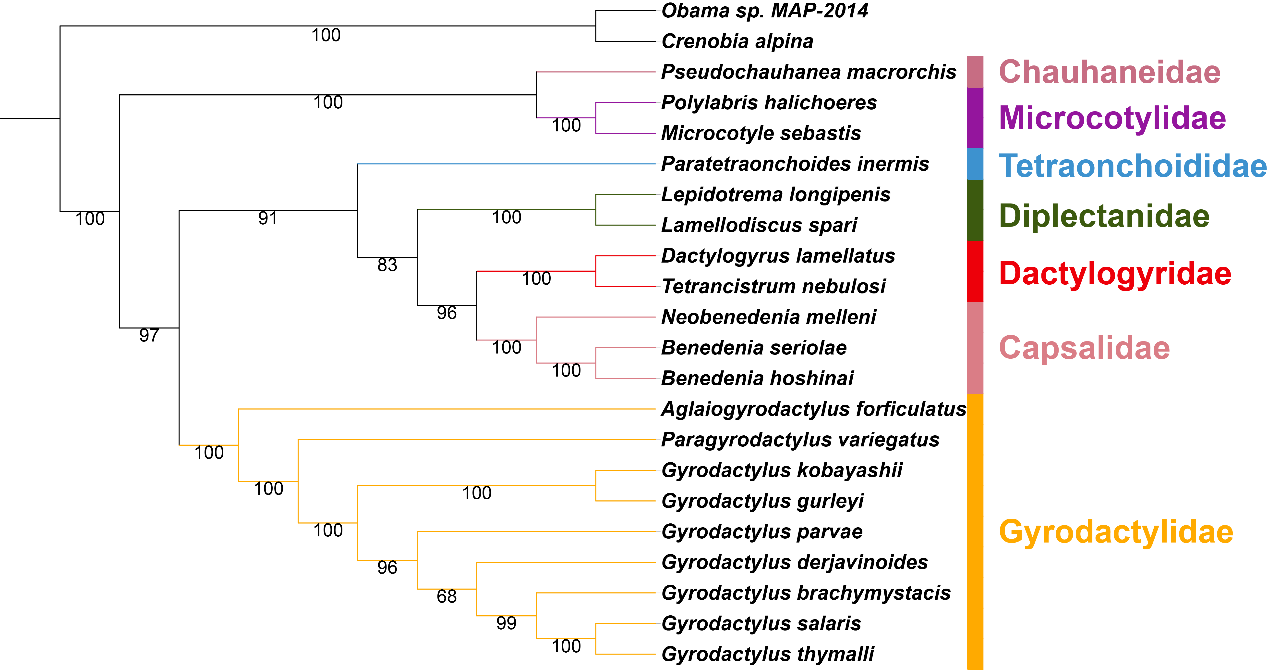


Figure S6 AliGROOVE analysis for three data sets. The obtained mean similarity score between sequences is represented by a colored square. Red indicates that ambiguously aligned sequence positions dominate between two sequences (i.e. heterogeneity), while blue indicates the opposite. PCGAA: amino acid alignment of 12 protein-coding genes; PCGNUC: codon-based alignment of 12 protein-coding genes; PCGRT: PCGNUC + nucleotide alignment of RNAs (22tRNA + 2rRNA).


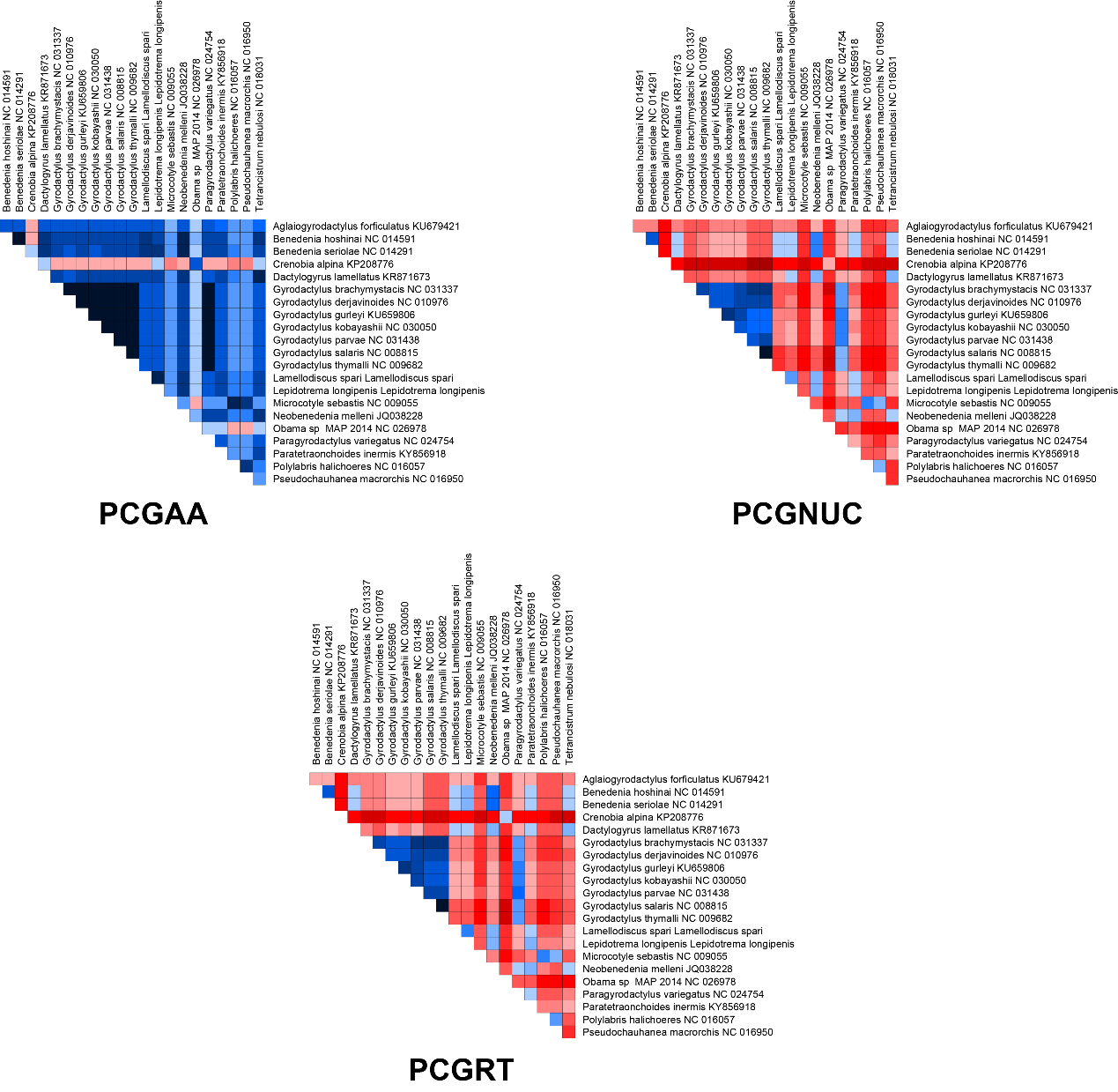


Table S3 The best partitioning scheme selected by PartitionFinder for PCGRT and PCGAA datasets (see Fig. S1 for abbreviations).

| **Dataset** | **Subset Partitions** | **Best Model** |
| --- | --- | --- |
| PCGRT | P1: (cox3_codon1, atp6_codon1) | GTR+I+G |
|  | P2: (atp6_codon2, nad3_codon2, nad4_codon2) | GTR+I+G |
|  | P3: (nad4L_codon3, atp6_codon3) | GTR+G |
|  | P4: (cox1_codon1) | GTR+I+G |
|  | P5: (cox1_codon2) | GTR+I+G |
|  | P6: (cox1_codon3, nad1_codon3) | GTR+I+G |
|  | P7: (cox2_codon1) | GTR+I+G |
|  | P8: (cox3_codon2, cox2_codon2) | GTR+I+G |
|  | P9: (cox2_codon3, cytb_codon3) | GTR+I+G |
|  | P10: (cox3_codon3, nad3_codon3) | GTR+I+G |
|  | P11: (cytb_codon1) | GTR+I+G |
|  | P12: (cytb_codon2) | GTR+I+G |
|  | P13: (nad1_codon1) | GTR+I+G |
|  | P14: (nad1_codon2) | GTR+G |
|  | P15: (nad2_codon1) | GTR+I+G |
|  | P16: (nad2_codon2) | GTR+I+G |
|  | P17: (nad2_codon3, nad6_codon3, nad4_codon3, nad5_codon3) | GTR+I+G |
|  | P18: (nad5_codon1, nad4_codon1, nad3_codon1) | GTR+I+G |
|  | P19: (nad4L_codon1, nad6_codon1) | GTR+I+G |
|  | P20: (nad4L_codon2) | GTR+G |
|  | P21: (nad5_codon2, nad6_codon2) | GTR+I+G |
|  | P22: (rrnS, rrnL) | GTR+I+G |
|  | P23: (tRNAs) | GTR+I+G |
| PCGAA | P1: (atp6, cox2, cox3, cytb) | VT+I+G+F |
|  | P2: (cox1) | MtArt+G |
|  | P3: (nad1, nad2, nad3, nad4L, nad4, nad5, nad6) | MtArt+I+G+F |
